# New Appliances and Things Medical

**Published:** 1899-09-30

**Authors:** 


### NEW APPLIANCES AND THINGS MEDICAL.

[We shall be glad to receive, at our Office, 28 & 29, Southampton Street, Strand, London, W.C., from the manufacturers, specimens of all new preparations and appliances which may be brought out from time to time.]

### CONSUMPTION CHART AND CASE PAPER.

(J. WRIGHT AND CO., BRISTOL.)

THE first of these is intended for the use of the nurse, and is, in fact, a temperature chart arranged more especially for cases of consumption, providing spaces, for example, in which to record details as to sputum, hæmoptysis, chest measurement, and other matters which have to be taken note of in such cases. The case paper is apparently for the use of the medical attendant. It is a foolscap sheet so ruled and heade as to leave appropriate spaces for many of the points to which inquiry is directed in investigating a case of phthisis, and having in addition a couple of outline diagrams of the front and of the back of the chest in which to record the physical signs. As the tendency now is to treat consumption in separate hospitals, or, at any rate, in separate wards, these special charts and case papers will probably be found very useful as tending to systematise the observations which have to be recorded in regard to the cases.

